# Natural Phytochemicals as SIRT Activators—Focus on Potential Biochemical Mechanisms

**DOI:** 10.3390/nu15163578

**Published:** 2023-08-14

**Authors:** Michał Wiciński, Jakub Erdmann, Agnieszka Nowacka, Oskar Kuźmiński, Klaudia Michalak, Kacper Janowski, Jakub Ohla, Adrian Biernaciak, Monika Szambelan, Jan Zabrzyński

**Affiliations:** 1 Department of Pharmacology and Therapeutics, Faculty of Medicine, Collegium Medicum in Bydgoszcz, Nicolaus Copernicus University, M. Curie 9, 85-090 Bydgoszcz, Polandkld.michalak@gmail.com (K.M.);; 2 Department of Neurosurgery, Faculty of Medicine, Collegium Medicum in Bydgoszcz, Nicolaus Copernicus University, M. Curie 9, 85-090 Bydgoszcz, Poland; 3 Department of Orthopaedics and Traumatology, Faculty of Medicine, Collegium Medicum in Bydgoszcz, Nicolaus Copernicus University, 85-090 Bydgoszcz, Poland

**Keywords:** sirtuins, phytochemical, resveratrol, curcumin, quercetin, fisetin, berberine, kaempferol

## Abstract

Sirtuins are a family of proteins with enzymatic activity. There are seven mammalian sirtuins (SIRT1-SIRT7) that are found in different cellular compartments. They are a part of crucial cellular pathways and are regulated by many factors, such as chemicals, environmental stress, and phytochemicals. Several in vitro and in vivo studies have presented their involvement in anti-inflammatory, antioxidant, and antiapoptotic processes. Recent findings imply that phytochemicals such as resveratrol, curcumin, quercetin, fisetin, berberine, and kaempferol may regulate the activity of sirtuins. Resveratrol mainly activates SIRT1 and indirectly activates AMPK. Curcumin influences mainly SIRT1 and SIRT3, but its activity is broad, and many pathways in different cells are affected. Quercetin mainly modulates SIRT1, which triggers antioxidant and antiapoptotic responses. Fisetin, through SIRT1 regulation, modifies lipid metabolism and anti-inflammatory processes. Berberine has a wide spectrum of effects and a significant impact on SIRT1 signaling pathways. Finally, kaempferol triggers anti-inflammatory and antioxidant effects through SIRT1 induction. This review aims to summarize recent findings on the properties of phytochemicals in the modulation of sirtuin activity, with a particular focus on biochemical aspects.

## 1. Introduction

In the last few years, due to an increasingly aging society and deteriorating eating habits, scientists have been looking for substances that exert a positive impact on the lives and health of humans. Sirtuins are a family of proteins engaged mainly in physiological processes and metabolic pathways, which implies that they are a promising target of research. Chemicals, phytochemicals, environmental stress, and other factors can regulate the activity of sirtuins [1]. Plants and plant extracts, due to their phytochemical content, have been used as sources of medicine since ancient times. Natural phytochemicals are primary or secondary organic metabolites produced by plants and fungi. Primary metabolites regulate processes crucial for plants (growth, development, reproduction, and metabolism), whereas secondary metabolites are involved in the interaction of plant species, such as competition and protection from damage and diseases. Phytochemicals are classified into three groups based on their biosynthetic origins: terpenoids, phenolic compounds, nitrogen-containing alkaloids, and sulfur-containing compounds [2]. Recent findings suggest that many biochemical mechanisms in human cells might be regulated by natural phytochemicals that can be beneficial for health preservation, disease prevention, and general homeostasis. Phytochemicals have numerous properties such as anti-inflammatory, anticancer, antibacterial, anti-allergic, analgesic, and antioxidant [3]. So far, extensive research has been conducted regarding the supplementation of sirtuins activators, including phytochemicals, and their role in homeostasis maintenance.

Sirtuins are a class of III NAD-dependent histone deacetylases and can be found in the cells of many species, from yeast to mammals. They are homologs of yeast silent information regulator 2 (Sir2), the first identified sirtuin [4,5]. Sirtuins are known to be part of crucial cellular pathways, such as PI3K/AKT, AMPK, and mTOR, which regulate the balance between anabolic and catabolic processes, including inflammation, oxidation of fatty acids, DNA repair, histone deacetylation, insulin sensitivity, oxidant-antioxidant balance, aging, mitochondrial function, and many others [6,7]. Sirtuins might have a significant influence on the pathogenesis of a wide range of diseases [5,8]. In mammalian cells, the sirtuin family is represented by seven enzymes (from SIRT1 to SIRT7), whose main activity is deacetylation. Sirtuins are located in different tissues and subcellular compartments and are associated with a variety of functions in cellular metabolism. SIRT1 and SIRT2 localize in the nucleus and cytoplasm; SIRT3, SIRT4 and SIRT5 are found in mitochondria; and SIRT6 and SIRT7 mostly reside in the nucleus and nucleoli [8,9]. Changes in the physiological state of the cell caused by noxious stimuli are among the factors causing transcriptional and post-transcriptional adjustments in the level of sirtuins. For instance, stimuli causing cellular stress such as hypoxia, inflammation, or DNA damage increases the level of SIRT1 by binding its promoter to transcription factors HIF-1α, NFĸB, and E2F1, respectively [10,11].

It is believed that phytochemicals, as easily accessible compounds, might help improve quality of life during aging. In this article, we discuss how phytochemicals can activate proteins from the sirtuin family and regulate many metabolic pathways that participate in key cell processes. 

## 2. SIRT-1

Sirtuin 1 (SIRT1) is the most studied and well-described member of the sirtuin family. SIRT1 causes deacetylation of histones (H1, H3, and H4) and many other proteins, including transcription factors [8]. SIRT1 plays a crucial role in protecting against oxidative stress. Modulation of transcription factors such as PPAR (α and γ), NRF, and TFAM increases antioxidant responses and regulates mitochondrial functions. Furthermore, SIRT1 regulates forkhead box O (FOXO) proteins that are involved as transcription factors in the regulation of many cellular pathways affecting longevity, cellular differentiation, growth regulation, stress resistance, and apoptosis. In mammals, FOXO1, FOXO3, FOXO4, and FOXO6 genes are found [12]. Deacetylation of FOXO3a by SIRT1 causes modulation of antioxidant enzymes: superoxide dismutase 2 (SOD2) and catalase (CAT) [5,13]. SIRT1 regulates acetylation of NFĸB and therefore modifies transcription of genes associated with inflammatory factors: IL (interleukin)-1, IL-8, IL-6, TNF-α and others [13,14]. It also stimulates deacetylation of peroxisome proliferator-activated receptor gamma coactivator 1-alpha (PGC-1α) and endothelial nitric oxide synthase (eNOS), which increases their activity and leads to inhibition of NF-kB transcription [15]. Suppression of NF-kB by nitric oxide (NO) derived from eNOS results in reduced oxidative stress through PGC-1α/Nrf1 system sensitization. Due to the mentioned effect, research suggests the essential role of SIRT1 in maintaining a healthy cardiovascular system [16].

It is known that 5′ AMP-activated protein kinase (AMPK) and SIRT1 can activate each other and share many common target molecules. Research has demonstrated that decreased SIRT1 protein activity and decreased AMPK phosphorylation degree may cause the absence of the stimulating effect of insulin on glucose uptake [17]. A study has been performed on primary rat podocytes cultured in standard or high glucose conditions. Decreased SIRT1/AMPK activity under hyperglycemia conditions was considered as a cause of higher insulin resistance. Improved glucose uptake in podocytes was achieved by S-nitroso-N-acetylpenicillamine (NO donor) treatment, preventing a high glucose-induced decrease in SIRT1 and AMPK activity and increased GLUT4 protein expression [18,19]. GLUT4 is an insulin-dependent glucose transporter that allows the passage of glucose from intracellular storage depots to the plasma membrane [20]. AMPK is also involved in insulin-dependent remodeling of the actin cytoskeleton and GLUT4 translocation from cytoplasm to the cell membrane. Insulin resistance is a component of metabolic syndrome and is important in the pathogenesis of many diseases [21]. The participation of SIRT1 in many cellular pathways associated with the maintenance of glucose homeostasis indicates its important protective role in metabolic syndrome.

## 3. SIRT-2

Sirtuin 2 (SIRT2) is a predominantly cytosolic sirtuin, but it is also found in the mitochondria and nucleus. As one of the seven mammalian isoforms of sirtuins, SIRT2 is the least understood [22]. SIRT2, with deacetylation as the main activity, participates in cell cycle regulation (by deacetylation of H4K16) and oxidant-antioxidant homeostasis. The expression of antioxidant enzymes is increased after deacetylation of PCG-1α by SIRT2. This results in a reduction of reactive oxygen species (ROS) levels and protection against oxidative damage [5,11]. Oxidative stress is a component of many diseases, including atherosclerosis, chronic obstructive pulmonary disease, Alzheimer’s disease (AD), and cancer [23]. Cell cycle regulation might be initiated by BubR1 SIRT2-deacetylation [24]. BubR1 is a mitotic checkpoint kinase. There are reports that deacetylation of BubR1 lysine 668 inhibits ubiquitylation and the targeting of BubR1 to the proteasome [25]. A study has shown that the levels of BubR1 are reduced with age due to a decline in NAD+ and the ability of SIRT2 to maintain lysine-668 of BubR1 in a deacetylated state. Mice with overexpression of BubR1 live longer, whereas mice hypomorphic for BubR1 show signs of accelerated aging. On the other hand, some studies have reported that SIRT2 accumulation in the central nervous system with aging may be a negative sign. Inhibition of this process appears to be protective against aging-related neurogenerative diseases [26]. This effect was explained by SIRT2-dependent deacetylation of the FOXO3a transcription factor, which is closely related to aging, oxidative stress and apoptosis. Decreased oxidative stress and pro-apoptotic protein levels were observed when SIRT2 activity and FOXO3a expression were inhibited [26]. However, another study presented a positive effect of FOXO3a SIRT2-deacetylation in a response to oxidative stress by an increased level of mitochondria-localized manganese superoxide dismutase (MnSOD) [22]. It shows that the role of SIRT2 in regulating the oxidative stress response is not clear, and further research is necessary.

## 4. SIRT-3

Sirtuin 3 (SIRT3) is located mainly in the mitochondrial matrix of cells with high metabolic activity and possesses the function of deacetylase [9]. SIRT3 activates enzymes of various mitochondrial pathways associated with energy metabolism, such as glycolysis, oxidation of fatty acids, tricarboxylic acid (TCA) cycle, oxidative phosphorylation, synthesis of ketone bodies, urea cycle, amino acids catabolism, and mitochondrial protein synthesis. In the TCA cycle, deacetylation is mediated by SIRT3, which activates components of the electron transport chain complex (NDUFA9, SDHA). SIRT3 also participates in the regulation of ATP synthase activity and the deacetylation of isocitrate dehydrogenase 2 (IDH2), which is crucial for the TCA cycle due to its ability to promote the oxidation of isocitrate to α-ketoglutarate, resulting in NADPH production. The variety of metabolic pathways mediated by SIRT3 proves its major role in the energy homeostasis of cells [27,28].

Oxidative stress associated with ROS production leads to mitochondrial DNA mutation, damage to proteins and lipids, and severe nuclear DNA damage, causing the death of the cell [29]. Activation of SIRT3 in multiple ways enhances the ability of cells to manage ROS produced mainly in the mitochondrial electron transport chain (ETC). SIRT3 directly regulates SOD2 by deacetylation and inhibits the production of superoxide radicals. Furthermore, the abovementioned activation of enzyme IDH2 by SIRT3 leads to the replenishment of the NADPH pool necessary for the glutathione reductase metabolic pathway, the part of the antioxidant system that is important for cells under oxidative stress [28,30].

## 5. SIRT-4

Sirtuin 4 is mostly located in the mitochondrial matrix and presents the activity of deacetylase, ADP-ribosyl-transferase, lipoamidase, and deacylase [31]. Studies that have focused on the association between SIRT4 activity and tumorigenesis emphasize the role of SIRT4 as a tumor suppressor [32]. In cells of human cancers such as pancreatic, gastric, ovarian, renal, prostate, liver, or lung cancer, decreased levels of SIRT4 mRNA are observed [33]. Tumor suppressive properties presented by SIRT4 are associated with inhibition of glutamine catabolism caused by DNA damage. In DNA-damaging conditions, the upregulation of SIRT4 promotes the self-protection of cells by inhibiting restorations of TCA. TCA is necessary for glutamine catabolism, which provides the energy required for cancer cells to proliferate [34].

SIRT4 is a significant regulator of lipid homeostasis. It modulates fatty acid oxidation in white adipose tissue and skeletal muscle by deacetylation and inhibition of mitochondrial malonyl-CoA decarboxylase (MCD). The conversion of malonyl-CoA into acetyl-CoA is interrupted by the adeacetylated form of MCD, which leads to the accumulation of malonyl-CoA. An increased level of malonyl-CoA inhibits fatty acid oxidation and promotes fat synthesis [33,35]. Furthermore, SIRT4 affects lipid synthesis by controlling the expression of genes associated with fat metabolism. The level of hepatic peroxisome proliferator-activated receptor α (PPARα) expression may be controlled by SIRT4, resulting in inhibition of genes responsible for lipid catabolism. SIRT4 also decreases the fat oxidative capacity of hepatic cells by inhibiting fatty acid oxidation genes expression [36].

## 6. SIRT-5

The last of the mitochondrial sirtuins, sirtuin 5 (SIRT5), is localized mainly in the mitochondrial matrix and presents the activity of demalonylase, desuccinylase, and deacetylase. It is involved in many cellular processes, including fatty acid oxidation, the formation of ketone bodies, apoptosis, and the urea cycle [9,37]. SIRT5 regulates mitochondrial acyl-CoA synthetase activity in liver tissues, and when its activity is decreased, it may lead to periportal liver steatosis [38]. The disease is manifested by triacylglycerol accumulation in the cytoplasm of hepatocytes [39]. It was shown that mice with the SIRT5 knockout gene developed macrovesicular steatosis after 5 weeks on a high-fat diet containing coconut oil. Furthermore, SIRT5 regulates ketogenesis through 3-hydroxy-3-methylglutaryl-CoA synthase (HMGCS2) succinylation changes. Decreased β-hydroxybutyrate production in vivo as well as increased accumulation of medium- and long-chain acylcarnitines were attributed to a lack of SIRT5 [40].

In the liver, SIRT5 controls ammonia detoxification through the regulation of the first enzyme of the urea cycle, carbamoyl phosphate synthase 1 (CPS1) [41,42]. CPS1, as a glutarylated protein, is targeted by SIRT5 for deglutarylation. Glutarylation of CPS1 inhibits cycle activity, but this process can be reversed by SIRT5 [43]. In cells with the SIRT5 knockout gene, ammonia is accumulated [41]. Moreover, SIRT5 is involved in the regulation of the oxidative stress response. Succinylation of SOD1 decreases its activity. SIRT5 is able to bind SOD1 and desuccinylate it, which allows it to maintain ROS balance in cells [44,45,46].

## 7. SIRT-6

Sirtuin 6 (SIRT6) plays a critical role in genomic stability. SIRT6, with ADP-ribosyl-transferase and deacetylase activity, is localized in the nucleus and participates in DNA repair by catalyzing PARP-1 mono-ADP-ribosylation. This leads to the stimulation of DNA double-strand break repair and enhances the stability of the genome and telomers [11]. Poly(ADP-ribose) polymerase (PARP) is an important enzyme involved in DNA damage signaling, chromatin remodeling, and repair [47]. Studies on mice with the SIRT6 knockout gene reported increased defects in DNA repair, a shortened lifespan, and a higher cancer incidence [48]. Furthermore, SIRT6 plays a role in DNA polymerase β (Polβ) and XRCC1 recruitment and PAR-poly(ADP-ribose) formation in response to DNA damage [49]. The significantly diminished recruitment of Polβ and XRCC1 to DNA damage was demonstrated in the absence of SIRT6. At the same time, SIRT6 is considered as a suppressor and promotor of diverse diseases such as cancer, diabetes, and inflammation. As an inhibitor of aerobic glycolysis, crucial for the growth of cancer cells, SIRT6 acts as a tumor suppressive factor. Hepatocellular, ovarian, lung, and breast cancers present decreased levels of SIRT6 and increased tumor progression. It may indicate that SIRT6 has a protective role in carcinogenesis. However, different studies show poor clinical prognosis related to the oncogenic function of SIRT6 in colon and hepatocellular cancers [50].

The role of SIRT6 is also controversial in ovarian cancer due to the fact that it is involved in the epithelial-to-mesenchymal transition of ovarian cancer cells. Ovarian cancer cell lines with the SIRT6 knockout gene inhibited migration and invasion without affecting proliferation [51].

Recent studies have shown that it may inhibit the progression of vascular calcification associated with chronic kidney disease [52]. SIRT6 has the ability to inhibit the osteogenic transdifferentiation of vascular smooth muscle cells through the downregulation of runt-related transcription factor 2 (Runx2). Runx2, due to SIRT6 activity, is degraded by the ubiquitin-proteasome system.

## 8. SIRT-7

Sirtuin 7 (SIRT7) is primarily localized in the nucleus, specifically in the nucleoli, and possesses the activities of deacetylase, desuccinylase, and deglutarylase. It plays a significant role in chromatin remodeling, rDNA transcription, the metabolism of lipids, age-related processes, and many others [4]. A decreased level of SIRT7 is associated with aging processes in tissues and organs (liver, lungs, colon, skin, heart, hair follicles, and blood) [53]. Studies showed that mice with SIRT7 deficiency suffer from disorders related to aging (i.e., reduced bone mass) and have a shortened lifespan [53,54]. The anti-aging mechanism of SIRT7 was also observed in human stem cells and was based on the formation of complexes with heterochromatin proteins and nuclear lamina. This connection maintains heterochromatin in a repressive state and affects the immune response with ageing. What is more, SIRT7 also protects the stem cells of hair follicles from aging by activating the telogen-to-anagen transition. Deacetylation of NFATc1 activates follicle stem cells and promotes the progression of the hair growth cycle [53,55].

**Figure 1:**
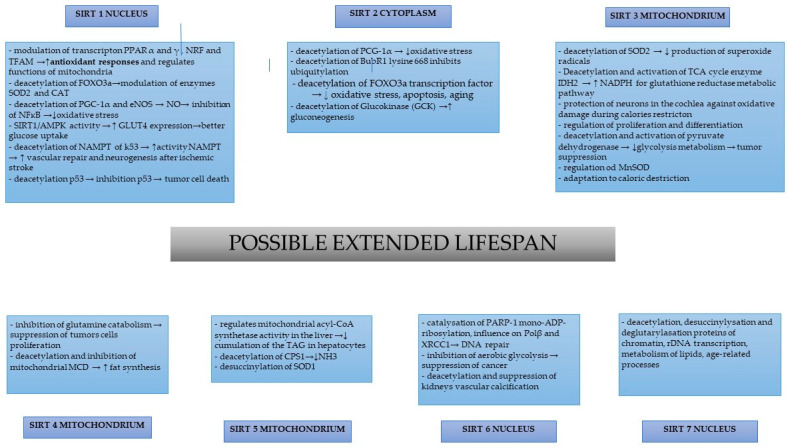
Sirtuins and potential biochemical processes that may delay cellular senescence and prolong the organismal lifespan.

A summary of the studies describing the activity of particular sirtuins is shown in Figure 1.

## 9. Natural Phytochemicals as Sirtuin Activators

Bioactive compounds can act as antioxidants and anti-inflammatory agents, reducing the negative effects of oxidative stress and the incidence of chronic diseases. Many studies have proven that polyphenols, flavonoids, some free fatty acids, and polysaccharides are compounds that can modulate sirtuins pathways [56]. Molecules referred to as natural phytochemicals can also modulate sirtuin activity [57,58] Bioactive sirtuin-modulating compounds from natural sources can be found in many dietary products, such as fruits, vegetables, herbs, spices, olive oil, wine (especially red), and tea [58,59]. A summary of phytochemical’s sources is shown in Table 1.

## 10. Resveratrol

Resveratrol, also known as 3,5,4′-trihydroxy-trans-stilbene, is a naturally occurring polyphenol compound with a molecular weight of 228.2 g/mol, categorizing it as a small molecule. Its discovery dates back to 1940 when it was first isolated from the roots of *Veratrum grandiflorum* O. Loes, commonly known as white hellebore [61]. In addition to white hellebore, resveratrol can be found in various other plants, including grapes, cocoa, strawberries, tomatoes, peanuts, hop, cranberries, and sugar cane.

Resveratrol possesses the ability to directly bind and activate sirtuins, which are cellular sensors for NAD+. One of its crucial functions involves the regulation of SIRT1 and AMPK in a dose-dependent and reciprocal manner. At lower levels, resveratrol prompts SIRT1 to deacetylate and activate liver kinase B1 (LKB1), an upstream kinase of AMPK [62]. This leads to an increase in AMPK activity, which stimulates energy catabolism and elevates cellular NAD+ levels. Conversely, at higher levels, resveratrol enhances the cellular AMP-to-ATP ratio by potentially inhibiting mitochondrial ATP production [62]. This in turn activates AMPK. The activation of AMPK promotes autophagy and mitochondrial biogenesis by inhibiting mTOR, while SIRT1 inhibits the nuclear factor kappa-light chain enhancer of activated B cells (NF-κB), resulting in anti-inflammatory and anti-cancer effects. Resveratrol’s regulatory actions on SIRT1 and AMPK contribute to its potential anti-aging effects. Furthermore, resveratrol can activate AMPK through mechanisms involving cAMP and calcium, which positively impact aging and cellular senescence. By inhibiting phosphodiesterase (PDE), resveratrol raises the intracellular levels of cAMP and Ca^2+^, thereby activating the AMPK pathway. This pathway not only upregulates autophagy but also increases NAD+ levels, subsequently boosting SIRT1 activity [63]. Additionally, cAMP induces the expression of Nrf2, a transcription factor that promotes antioxidant gene expression.

Referring to the prevention of inflammation and oxidative stress, resveratrol, as a Nrf2 and SIRT1 activator, is shown to reduce the pathologic changes associated with aging in the kidneys. Those changes are based on the expression of senescence genes, changes in hormones, increases in oxidative stress, and damage to the mitochondria [64]. Kim et al. performed a study on male 18-month-old C57BL/6 mice. Resveratrol (40 mg/kg) was administered to the mice for 6 months. In this model of age-related renal injury, resveratrol enhanced renal function. Decreased proteinuria, attenuated glomerulosclerosis, reduced tubular interstitial fibrosis and inflammation cell infiltration were observed. Resveratrol-treated mice presented a higher expression of SIRT1/AMPK and PPARα compared to control mice [65].

Resveratrol, commonly referred to as RSV, has been closely associated with the “French Paradox”, which demonstrates the inverse relationship between red wine consumption and the occurrence of cardiovascular disease in the French population. The cardioprotective effects of red wine were initially reported in 1992, leading to numerous studies investigating the potential therapeutic benefits of resveratrol for various pathological conditions [66]. Over 110 clinical trials have been conducted to date, primarily focusing on resveratrol’s impact on cardiovascular functions and other biological processes [63]. Resveratrol has shown promising effects in slowing down the aging process and treating several diseases, including obesity, type 2 diabetes, cancer, cardiovascular diseases, neurodegenerative diseases, and immune system regulation [63,67,68,69]. However, conflicting results have emerged regarding its effects on energy metabolism in humans. For instance, clinical trials in humans showed the inconsistent impact of resveratrol on adipose tissue metabolism and gene expression. Furthermore, it was found that resveratrol upregulates the pentose phosphate and glycolytic pathways, which may suggest worsening insulin sensitivity [70]. Resveratrol acts on multiple pathways involved in nutrient sensing, energy metabolism, and epigenetic modulation, including the insulin/IGF-1, AMPK, mTORC1, and sirtuin pathways. Consequently, the mechanism of action underlying resveratrol’s potential anti-aging properties is complex [67].

As mentioned earlier, resveratrol directly activates sirtuins and indirectly activates AMPK. AMPK acts as a negative regulator of mTOR, thereby promoting autophagy and mitochondrial biogenesis. Impaired autophagy increases the risk of age-related neurodegenerative diseases such as Parkinson’s disease (PD) and AD [60]. Extensive studies in rodents have established the neuroprotective role of resveratrol in AD, Huntington’s disease, PD, and neurological injuries [71]. Oral administration of resveratrol in humans has demonstrated reductions in β-amyloid plaque, a marker of age-related brain changes and a contributor to AD [72]. These effects of resveratrol can be largely attributed to the activation of SIRT1 and AMPK.

In models of age-related eye diseases such as age-related macular degeneration and autophagy impairment, the accumulation of the autophagy receptor p62 has been observed. AMPK activity is associated with p62 accumulation, as AMPK phosphorylates and causes oligomerization of p62 during the formation of autophagosomes. Resveratrol can activate the AMPK pathway and promote autophagy, thereby reducing the accumulation of p62 and cellular waste, ultimately providing protection against age-related diseases such as macular degeneration. Furthermore, resveratrol can activate the SIRT1/PPAR γ co-activator 1 α pathway, enhancing mitochondrial function and proteostasis [73].

Several animal studies have demonstrated that resveratrol can prevent type 2 diabetes mellitus (T2DM), improve glucose metabolism, and enhance insulin sensitivity. For instance, mice fed a high-calorie diet and administered resveratrol orally showed improvements in insulin, glucose, IGF-1 levels, and the insulin sensitivity index compared to high-calorie diet controls [74]. These findings suggest that the activation of sirtuins and AMPK by resveratrol could be beneficial in preventing age-related diseases such as cardiovascular disease, T2DM, and neurodegenerative diseases.

The neuroprotective impact of resveratrol is attributed to its ability to promote neurogenesis and safeguard the central nervous system. Resveratrol has the capacity to traverse the blood–brain barrier via the circulatory system, leading to enhancements in antioxidant enzyme activity. Furthermore, it extends the effects of pathways associated with SIRT1 and induces glial activation, contributing to increased neurogenesis in the hippocampus. Additionally, resveratrol has been shown to reduce the expression of the amyloid precursor protein and enhance spatial working memory [22].

In terms of brain function, resveratrol complements the involvement of SIRT1/AMPK pathways by regulating gene expression in the hippocampus. In a study on diabetic rats induced by streptozotocin, resveratrol was found to balance the expression of genes related to neuronal development and synaptic plasticity (Hdac4, Hat1, Wnt7a, ApoE), as well as reduce pro-inflammatory signaling through Jak-Stat pathway (IL-15, IL-22, Socs2, and Socs5) [75]. Another study conducted on a rat model of vascular dementia demonstrated that resveratrol treatment increased the expression of nerve growth components in the hippocampus, leading to a decrease in pyramidal cell mortality in the CA1 region and improved spatial working memory [76]. Furthermore, resveratrol administration in rats with permanent bilateral carotid occlusion improved memory and learning abilities, as evidenced by the Morris water maze test. Resveratrol reduced oxidative stress markers (malonyldialdehyde) and increased the levels of antioxidant enzymes (superoxide dismutase) and glutathione in the cortex and hippocampus [77].

Recent studies have also shed light on the effects of resveratrol on the injured brain. It has been shown to attenuate the inflammatory response and alleviate traumatic brain injury by reducing ROS production and inhibiting the activation of NLRP3 inflammasome. These effects may be mediated through the involvement of SIRT1 [78]. Resveratrol has also been found to increase the expression of genes encoding antioxidants and anti-aging factors (SIRT1 and SIRT3) in AD patients [79]. In neonatal hypoxic-ischemic brain injury, resveratrol activates SIRT1 to inhibit HMGB1/TLR4/MyD88/NF-kB signaling and subsequent neuroinflammatory responses, providing neuroprotective effects [80]. Additionally, resveratrol protects against cognitive impairment induced by chronic unpredictable mild stress by activating the SIRT1/miR-134 pathway, which leads to increased expression of CREB and BDNF in the hippocampus [81]. Lastly, resveratrol has been shown to mitigate learning and memory impairments in juvenile animals fed a high-caloric diet, potentially through the upregulation of p16 or the downregulation of PPAR in the hippocampal CA1 region [82].

Besides all the above, resveratrol has also the potential to protect against ovarian aging by engaging cellular mechanisms associated with SIRT1, which helps safeguard oocytes from age-related impairments [83]. In rat granulosa cells, resveratrol was found to increase the expression of SIRT1, LH receptor, StAR, and P450 aromatase genes, while the mRNA levels of FSH receptor remained unaffected [84]. This indicates that resveratrol and SIRT1 can regulate ovarian functions through the activation of molecules involved in folliculogenesis and gonadotropin receptors. In swine granulosa cells, resveratrol dose-dependently elevated SIRT1 mRNA and protein levels, leading to an acceleration of cell apoptotic rate and follicular atresia [85]. Supplementation of resveratrol in cultured porcine ovarian granulosa cells resulted in increased SIRT1 protein levels and apoptosis, promoting the release of testosterone and estrogen while inhibiting cell proliferation [86]. When added to the medium used for in vitro maturation (IVM), polydatin, a glycosidic form of resveratrol, improved embryo development by increasing SIRT1 protein levels and reducing ROS [26]. Additionally, in the same study, the protein levels of nuclear factor NF-kB and cyclooxygenase (COX2) were significantly lower in embryos cultured with polydatin. As NF-kB and COX2 are involved in inflammation, the authors suggested that the presence of polydatin may have a beneficial effect on embryo development by suppressing inflammatory processes [87]. In bovine granulosa cells, cumulus cells, oocytes, and blastocysts, SIRT1 was detected through immunofluorescence and Western blot analysis. Furthermore, resveratrol increased SIRT1 mRNA and protein levels in cumulus cells, suggesting that the positive effects of resveratrol on oocyte maturation and embryonic development during in vitro fertilization may be mediated by SIRT1 [88].

Cai et al. recently demonstrated the role of resveratrol in protecting ovarian granulosa-lutein cells (GLCs) against hydrogen peroxide (H_2_O_2)_ in 3-week-old female Sprague Dawley rats. H_2_O_2_ in GLCs decreases cell viability, impairs cellular morphology, reduces levels of progesterone and estradiol, and intensifies cell apoptosis by decreasing the level of anti-apoptosis protein Bcl-2 and increasing the level of pro-apoptosis protein such as Bax. The positive impact of resveratrol in GLCs was based on the SIRT1/Nrf2 pathway activation, which led to increased levels of antioxidant enzymes and augmented antioxidant capacity to resist oxidative stress [89].

A summary of the studies describing the impact of resveratrol on sirtuins is shown in Table 2.

## 11. Curcumin

Curcumin, scientifically known as 1,7-bis(4-hydroxy-3-methoxyphenyl)-1,6-heptadiene-3,5-dione or diferuloylmethane, is a natural compound classified as a diarylheptanoid and a member of the curcuminoid group. It is derived from the root of Curcuma longa, commonly referred to as turmeric. Extensive research has demonstrated that curcumin possesses a range of beneficial properties, including anti-aging, antioxidant, anti-inflammatory, antihypertensive, anti-diabetic, anti-obesity, antiapoptotic, and cancer-preventive activities [90,91,92]. Curcumin is extracted from Curcuma longa and is utilized not only as a dietary supplement but also as a condiment or coloring agent in diverse culinary and textile applications worldwide. Within the turmeric extract, curcumin typically represents 60–75% of the composition, with the remaining fraction consisting of its analogues, namely demethoxycurcumin and bisdemethoxycurcumin [67,93].

Curcumin has been found to possess the ability to modulate the activity of sirtuins, thereby facilitating cellular adaptation to various conditions, including stress factors. This characteristic places curcumin in the category of hormetins [94]. Research has centered on exploring the dose-dependent biphasic effects of curcumin on skin fibroblasts [95], demonstrating its adaptative properties. Studies using replicative senescence and premature senescence models with doxorubicin have shown that at low levels of exposure (0.1–1 μM), curcumin does not prolong biological aging but instead up-regulates sirtuins in vascular smooth muscle cells (VSMCs) and endothelial cells. Conversely, higher concentrations of curcumin (2.5–10 μM) trigger senescence in cancer and endothelial cells [88,89]. Thus, curcumin demonstrates advantageous, protective, and anti-aging effects at lower concentrations, while higher concentrations may lead to cytotoxic and pro-senescent effects. Endothelial cells seem to exhibit greater vulnerability to these effects when compared to VSMCs, possibly attributed to their unique capacity to enhance sirtuin levels [95,96,97]. Moreover, studies have demonstrated that the downregulation of SIRT7 by curcumin in VSMCs can induce anti-proliferative effects, suggesting its potential in alleviating cardiovascular conditions [98]. The elevation of sirtuins plays a role in the observed anti-aging effects of curcumin in multiple experimental studies. For instance, curcumin has been shown to improve the differentiation ability of keratinocytes in a senescence model [99]. In the nematode Caenorhabditis elegans, curcumin prolonged lifespan, and this outcome was nullified by mutations in the SIRT2 gene [100].

SIRT1 plays a pivotal role in regulating the transcription and expression of inflammatory markers such as IL-1, TNF-α, IL-8 and IL-6. This is achieved through the acetylation of NF-κB and p65 [101]. By interacting with the JAK/STAT pathway, suppressor of cytokine signaling (SOCS) and TLR4/MyD88/NF-κB axisSIRT1 influence inflammatory pathways [102]. For instance, curcumin is known to suppress STAT3 phosphorylation, which leads to inhibition of the inflammatory network. Furthermore, in both in vitro and in vivo, it promotes the expression of SOCS1 and SOCS3, which serve as antagonists of the JAK/STAT pathway and eventually inhibits the release of TNF-α, IL-6, and PGE2 [13,102,103].

The positive results of curcumin’s anti-inflammatory effects have been linked to its beneficial impact in numerous inflammatory conditions. For instance, in a mouse model of acute lung injury, the administration of curcumin led to a decrease in NF-κB expression and an increase in SIRT1 levels, resulting in a favorable outcome [104]. Similarly, in rat models of hemorrhagic shock after lung injury or chronic obstructive pulmonary disease (COPD), curcumin exhibited positive effects on pulmonary barrier function, oxidative stress reduction and attenuated lung inflammation, and the promotion of autophagy while inducing SIRT1 activity [105,106]. In another rat model of COPD, curcumin reduced oxidative stress and inflammation markers while increasing the expression of SIRT3 [107]. Furthermore, curcumin presented protective capabilities in a rat model of aluminum phosphide-induced lung injury by decreasing oxidative stress and increasing the expression of SIRT1, FOXO1, and FOXO3 [108]. Similar positive results were observed in lymphocytes isolated from COPD subjects, where curcumin increased SIRT1 expression, reduced steroid resistance, and decreased the release of IFNγ and TNF-α. However, the last effect may be achieved when curcumin is applied in combination with prednisolone [109].

Curcumin’s effects extend beyond its anti-inflammatory role. In rat chondrocytes treated with tertbutyl hydroperoxide, curcumin stimulated SIRT1 expression, inhibited endoplasmic reticulum stress, and contributed to the inhibition of osteoarthritis progression in animal models [110]. In the colon tissue of mice with dextran sulfate sodium-induced ulcerative colitis, curcumin exhibited protective effects by stimulating mTOR phosphorylation and SIRT1 expression, resulting in reduced body weight loss and severity of illness [111]. Moreover, in a neonatal rat model of necrotizing microscopic colitis, curcumin triggered the activation of the SIRT1/Nrf2 pathway, decreased TLR4 expression, and delayed disease progression [112].

Curcumin’s antioxidant effect is also associated with its modulation of sirtuin expression. In a rat model of cisplatin-induced renal impairment, curcumin triggered the activation of SIRT1, SIRT3, and SIRT4, reducing oxidative stress and protecting the kidneys from abnormal changes [113,114]. Furthermore, in a rat model of male infertility, curcumin reduced testicular oxidative stress and inflammation caused by cyclosporine use by enhancing SIRT1 expression [115]. In a mouse model of sepsis-induced acute kidney injury, tetrahydrocurcumin elevated survival rates, improved kidney function, reduced inflammatory response and oxidative stress, and prevented cell apoptosis through increased SIRT1 expression [116]. Curcumin also exhibited protective effects in a rat model of gentamicin-induced acute kidney injury by reducing tubular cell apoptosis, oxidative stress, and inducing SIRT1 and Nrf2/HO-1 expression [117].

Curcumin has been shown to increase SIRT3 expression, decrease superoxide dismutase activity, protect against oxidative stress, and suppress iron loading-induced autophagy in mouse and cell models of iron overload [118].

In studies using a mouse model of diabetes, curcumin administration modulated SIRT1 through AMPK, leading to improved glucose absorption and metabolism [119]. Curcumin protected vascular smooth muscle cells by activating AMPK, promoting ATP and superoxide synthesis, and enhancing SIRT1 activity. Furthermore, in models of myocardial infarction/reperfusion injury, curcumin effectively decreased oxidative stress-induced damage to mitochondria reduced infarction size through a SIRT1 pathway [120]. Moreover, conducted studies on cardiomyocytes exposed to hypoxia/reoxygenation cycles revealed curcumin’s capacity to diminish apoptosis and oxidative stress while increasing SIRT1 expression [121]. Additionally, in a streptozotocin-induced diabetes mouse model, tetrahydrocurcumin enhanced cardiac parameters, reduced myocardial fibrosis and cardiac hypertrophy, diminished ROS generation, and elevated SIRT1 expression [122]. In a mature mouse model of coronary artery ligation, curcumin administration led to enhancedSIRT1 expression and exhibited an anti-fibrotic effect [123]. Curcumin also mitigated age-related impairments in endothelial cells and vascular smooth muscle cells through the SIRT1 pathway and NF-κB inhibition [123,124].

These diverse effects of curcumin may have implications for various pathologies such as diabetes, cardiac fibrosis, or ischemia/reperfusion injury. Experimental studies have shown that curcumin decreases foam cell formation and intracellular lipid accumulation, facilitates cholesterol efflux in macrophages by activating SIRT6, and triggers the AMPK-SIRT1-LXRα signaling pathway, thus exhibiting its anti-atherosclerotic properties [125,126]. Long-term administration of curcumin in a mouse model of atherosclerosis induced by a high-fat diet protected against decreased SIRT1 expression, senescent cell accumulation, and vascular inflammation [127].

Curcumin, a natural compound, exhibits neuro-hormetic properties and enhances the adaptive stress response in a dose-dependent manner. It is achieved by increased expression of various factors such as antioxidant enzymes, sirtuins, FOXO transcription factors, chaperones, neurotrophic factors, and anti-apoptotic proteins. As a result, curcumin suppresses the progression of pathological mechanisms in both animal and cell models of neurodegenerative disorders such as AD and PD. The neuroprotective effects of curcumin are achieved through the modulation of potential factors such assirtuins, PGC-1α, oxidative stress, inflammation, and mitochondrial dysfunction that are associated with neuronal damage [128]. In AD, curcumin in combination with other natural compounds, has shown promise in reducing hallmark indicators of neuronal damage and slowing down cognitive impairment. It can regulate amyloid deposits, neurofibrillary tangles, synaptic loss, inflammation, autophagy, and oxidative stress. Many of these effects have been linked to an increase in the expression of sirtuin proteins [129]. Curcumin has been observed to display antioxidant effects in the hippocampus of mature rats, which have been associated with elevated levels of SIRT2 [130].

Curcumin has also shown other neuroprotective effects. It attenuates loss of memory in models of amyloid-induced neuronal metabolic dysfunction and improves cognition in transgenic mice by facilitating SIRT3 activity [131]. It improves mitochondrial membrane potential and inhibits apoptotic cell death in neurons treated with Aβ25-35 peptide. Curcumin activates SIRT1 expression and decreases the expression of Bax, a protein involved in apoptosis, in the presence of Aβ25-35 [132]. Moreover, curcumin reduces glutamate excitotoxicity in cultured neurons by increasing SIRT1 expression and reducing the level of acetylated PGC1α through SIRT1-mediated deacetylation [133]. In a rat model of cerebral ischemia/reperfusion injury, curcumin decreases inflammatory markers, induces SIRT1 and Bax expressions, and exhibits clear neuroprotective effects [134]. Bisdemethoxycurcumin, a derivative of curcumin, also demonstrates neuroprotective effects in experimental models of AD. These effects are connected to enhanced SIRT1 activity and the regulation of GSH and SOD levels [135].

Curcumin’s impact on sirtuins has been observed in clinical studies. In a randomized, double-blind clinical study involving patients with polycystic ovary syndrome (PCOS), curcumin administration resulted in reduced oxidative stress markers, increased gene expression of PGC1-α, and a non-significant increase in gene expression of SIRT1, along with enhanced SOD enzyme activity [136]. Similar results were found in animals, where female mice treated with curcumin exhibited elevated expression of SIRT1 and SIRT3, associated with improved follicle number, oocyte maturation, fertilization, embryo development, and decreased oxidative stress [137].

Studies have demonstrated the anti-cancer potential of curcumin, particularly in inducing apoptosis in cancer cells [124]. Experimental research has revealed that SIRT1 is overexpressed in colon cancer cells compared to normal cells. Inhibition of SIRT1 resulted in decreased viability and migration of cancer cells as well as a reduction in tumor volume and growth rate. Curcumin application has been shown to decrease the expression of SIRT1 protein in colon cancer cells and promote the proteasomal degradation of oncogenic SIRT1 [138].

Curcumin has the ability to activate SIRT1 pathways in all cellular compartments. Experimental and preclinical studies have indicated that SIRT1, as a cardioprotective factor, can be inhibited by stress factors associated with an increased risk of myocardial infarction. Reduced SIRT1 activity has been observed in inflammatory conditions and during the aging process, both of which are linked to oxidative stress. Hyperglycemia and hypercholesterolemia also inhibit SIRT1, but curcumin has been shown to restore its activity, acting as a protective agent against ischemia-reperfusion injury. Conversely, SIRT1 enhances mitochondrial processes, reduces oxidative stress, and mitigates inflammation in cardiomyocytes. Treatment with curcumin in cardiomyocytes exposed to hyperglycemia/osmotic stress has exhibited a protective effect, improving cell viability and enhancing SIRT3 expression and activity [139,140].

The cellular effects reported above are supported by animal studies. SIRT1 stimulation promotes insulin secretion while reducing glucagon secretion, leading to increased cellular glucose utilization and decreased blood glucose levels. Protective effects on mitochondrial metabolism have been observed in a mouse model of acute liver injury, where curcumin reduced oxidative stress and apoptosis by modulating hepatic SIRT1 activity [141]. Additionally, curcumin has been found to reduce inflammation and steatosis in a model of nonalcoholic fatty liver disease through an increase in SOD1 and SIRT1 expression [142]. In a hepatic steatosis model in postnatal overfed rats, dietary curcumin improved hepatic steatosis and mitochondrial function via SIRT3 activity [143]. Furthermore, curcumin induces a calorie restriction-type metabolic state by inducing AMPK, thereby exerting tumor-suppressing effects. These metabolic effects are mediated by SIRT1 induction, leading to a reduction in IL-6 and NF-κB levels and an increase in PPARα expression [144].

However, some of curcumin’s effects on sirtuin signaling pathways are still subject to debate due to inconsistent experimental and preclinical results. For instance, while curcumin has been shown to be a histone deacetylase inhibitor, studies have demonstrated that it does not significantly affect NLRP3 inflammasome activation mediated by histone deacetylase inhibition [145]. Conversely, other studies have demonstrated that curcumin promotes cell survival and mitochondrial quality in certain contexts by up-regulating PGC-1α and SIRT3, such as in bone marrow mesenchymal stem cells under hypoxic preconditioning and in wound healing processes [146]. Furthermore, curcumin has been found to support metabolic activity and muscle force gain in models of cancer-induced cachexia and disuse muscle atrophy, where increased SIRT1 activity is observed [147,148]. Additionally, curcumin has shown to improve muscle performance by stimulating SIRT1 activity [148]. Based on the available research curcumin has shown following biological effects on sirtuin proteins: activation of sirtuin-, modulation of gene expression, anti-inflammatory effects, oxidative stress and cellular survival, metabolic regulation. Effects of curcumin may vary and its used concentration can influence the outcome. The summary of the studies describing curcumin impact on sirtuins is shown in Table 3.

## 12. Quercetin

Quercetin (3,3′,4′,5,7-pentahydroxyflavone) is a representative of the natural flavonoid polyphenols. Its non-toxic properties exert a positive effect on antioxidant regulation and the treatment of aging-related diseases [13,149]. Quercetin also plays a beneficial role in anti-proliferative, anti-apoptotic, anti-inflammatory, and anti-cancer activities [150]. It is one of the most common flavonoids in the diet, found in glycoside form in fruits and vegetables such as apples, grapes, capers, onions, broccoli, soybeans, tea and nuts [151,152].

Quercetin is a substance with long lasting and strong anti-inflammatory properties in many tissues. Studies have demonstrated that quercetin, by targeting the activity of SIRT1, may regulate a wide spectrum of pathways, such as SIRT1/AMPK/NF-κB, SIRT1/Keap1/Nrf2/HO-1, and SIRT1/PI3K/Akt, which increase the activity of antioxidant enzymes and anti-inflammatory cytokines as well as the efficiency of mitochondrial processes [149]. For instance, quercetin inhibits oxLDL-induced mitochondrial dysfunction and ROS production through SIRT1 activation and NADPH/AKT pathway modification [153]. These properties of quercetin were also described in sepsis-induced C57BL/6 mice, but another biochemical explanation was given. Scientists found that quercetin decreased the mRNA expression of various factors, including C/EBP homologous protein (CHOP), 78-kDa glucose-regulated protein (GRP78), activating transcription factor 6 (ATF6) and increased activation of the AMPK/SIRT1 pathway. These changes resulted in the prevention of mitochondrial dysfunction and the suppression of oxidative stress [154]. Likewise, after quercetin treatment of non-small-cell lung cancer cells, the levels of SIRT1 and phosphorylated AMPK were substantially raised compared with the control group. The mentioned change induced pro-apoptotic autophagy, which may suggest quercetin anti-cancer properties [153]. What is more, Feng et al. presented interesting insights. Endoplasmic reticulum (ER) stress is connected with cell apoptosis, including activation of CHOP GRP78 and induction of the caspase pathway. The study conducted on male Sprague–Dawley rats suggested that the polyphenol decreases chondrocyte apoptosis by attenuating ER stress and downregulating the factors CHOP, GRP78, and caspase 3. All of the effects are believed to be achieved via the promotion of the AMPK/SIRT1 signaling pathway in vivo [110].

In very recent papers, researchers found that quercetin has potential for lowering the risk of developing type 2 diabetes [13,155]. The flavonoid via SIRT 1 activation reduces the sensitivity to T2D/insulin resistance in obese mice. Another described pathway is based on the reduction of NF-κB levels, which, consequently, diminishes oxidative damage in streptozotocin-induced diabetic rats [156]. Similar conclusions were presented by Ying et al., but in streptozotocin-induced broiler chicks [157].

The pharmacological properties of quercetin were also tested in ischemia-reperfusion injury. Tang et al. found that Sprague-Dawley rats with myocardial ischemia-reperfusion injury treated with quercetin had increased levels of SIRT1 and PGC-1α. The SIRT1/PGC-1α signaling pathway presented an anti-apoptic impact due to up-regulated Bcl-2 and down-regulated Bax expression [158]. A similar conclusion was stated by Leyton et al., but under different conditions. They conducted a study on Vero cells and the murine hippocampal neuronal cell line HT22 infected with HSV-1. The authors showed that quercetin inhibits *Herpes simplex* virus type 1-induced neurodegeneration through SIRT1 activation and subsequently inhibits proapoptotic transcription factors [159]. Moreover, other scientists performed a study on brain cells with ischemia-reperfusion injury and found decreased ROS production in the mitochondria via the SIRT1/NRF2/HO-1 pathway [160].

The abovementioned information shows that quercetin can be considered as a substance potentially beneficial for health in many ways. Polyphenol’s function is strictly connected with SIRT1 activation. Through different pathways, quercetin may reduce the production of ROS and mitigate mitochondrial dysfunction. In addition, it may influence carbohydrate metabolism and demonstrate antiapoptotic activity. A summary of the studies describing quercetin’s impact on sirtuins is shown in Table 4.

## 13. Fisetin

Another natural polyphenol known as fisetin (3,3′,4,7-tetra-hydroxyflavone) is a dietary flavonoid abundantly found in many fruits and vegetables such as strawberry, apple, persimmon, grape, onions, kiwi, and kale [162]. The flavonoid is known to present various beneficial effects such as anti-aging, anti-cancer, and anti-viral [163].

Research has described the involvement of fisetin in anti-obesity, anti-hyperlipidemic, and anti-diabetic biochemical mechanisms [163]. Several studies have reported that fisetin can activate the SIRT1 signaling pathway. Treatment with fisetin diminished the binding of PPARγ to the PPARγ promoter and simultaneously increased the binding of SIRT1 to the promoter. Fisetin possibly changes deacetylation and the activity of PPARγ by regulating the expression of SIRT1 as well as its affinity to the PPARγ promoter [162]. The enhanced association between SIRT1 and the PPARγ promoter inhibits adipogenesis and the accumulation of lipids [13]. Another mechanism of adipogenesis inhibition by fisetin was presented in 3T3-L1 cells. The increased level of SIRT1 led to the control of adipogenic transcription factors such as the CCAAT enhancer binding protein (C/EBP) family, peroxisome proliferator-activated receptor gamma (PPARg), Krüppel-like factors (KLFs), and sterol regulatory element-binding protein 1c (SREBP1c), which play crucial roles in the induction of adipocyte differentiation [162].

Recent studies found that fisetin enhances NF-kB deacetylation and the suppression of pro-inflammatory genes expression achieved via SIRT1 activation. These effects were observed on rat brains [164]. Another research presented the same biochemichal mechanisms and reported that treatment with fisetin prevented neuro-inflammation in induced- and natural-aging rat models by reducing the level of inflammatory markers [165]. Anti-inflammatory properties were also presented by Zheng et al. in human osteoarthritis (OA) chondrocytes [166]. They demonstrated that fisetin led to inhibition of IL-1β-induced inflammation as well as the degradation of Sox-9, aggrecan and collagen-II through SIRT1 activation. The authors also noticed that fisetin-supplemented mice had alleviated articular cartilage damage, subchondral bone sclerosis, and synovitis.

Interestingly, fisetin, as an activator of SIRT1, prevented germ cells from apoptosis caused by hydrogen peroxide. This effect was possibly achieved by the degradation of the transcription factor FOXO3a, which is the inductor of apoptosis [167]. The abovementioned studies allow us to imply that fisetin can lower the risk of oxidative stress-related degenerative diseases [168] and metabolic syndrome with its potential for the suppression of fat accumulation in obesity [162]. It also mitigates articular cartilage damage, which should be expanded in further studies. In all these effects, SIRT1 involvement is observed. A summary of the studies describing the impact of fisetin on sirtuins is shown in Table 5.

## 14. Berberine

Berberine (BBR) is an organic chemical compound, an isoquinoline alkaloid of plant origin, found in barberry and many other plants such as Hydrastis canadensis, Xanthorhiza simplicissima, Coptis chinensis, Tinospora cordifolia, Argemone Mexicana, and Eschscholzia californica [169]. This compound is characterized by its biological activity [170].

BBR has poor oral absorption [171] and low bioavailability due to first-pass effects in the intestinal lumen [172]. After oral administration, it undergoes complex metabolic processes, resulting in low plasma concentrations [173]. Its pharmacological effects are believed to be due to the activity of its metabolites. In vivo studies on rats have identified, among others, 54 berberine metabolites in urine and 39 in plasma [173].

BBR is used in the treatment of gastrointestinal, cardiovascular, and metabolic diseases. Its pleiotropic neuroprotective effects have been demonstrated. It also inhibits the proliferation of various types of cancer cells and improves the efficacy and safety of chemoradiotherapies [170].

In rats, the cardioprotective effect is related to the reduction of infarct size and the recovery of cardiac function after an ischemia/reperfusion injury (I/RMI) [174]. This mechanism is related to SIRT1 expression upregulation. Activation of SIRT1 is associated with increased expression of the antiapoptotic factor Bcl-2 and decreased expression in the proapoptotic factors Bax and caspase-3 [174]. Activation of SIRT1 in I/RMI also has antioxidant and anti-inflammatory effects by downregulating gp91^phox^ expression, reducing mRNA expression and the activity of interleukin-1β, interleukin-6, and tumor necrosis factor-α [175].

BBR has a lipid-lowering effect and reduces insulin resistance [169]. This type of activity is also the most documented in randomized clinical trials [176]. In one of the studies on rats on a high-fat diet with induced insulin resistance, the effect of BBR on hepatic metabolism with the participation of the PI3K/Akt-p/SIRT1/PTEN pathway was demonstrated [177]. This study confirmed that the activation of SIRT1 together with AMPK is essential in increasing the level of fatty acid metabolism, and that SIRT1 affects the insulin signaling pathway by regulating insulin secretion from β-cells via the regulation of UCP2 expression and influencing the depolarization of these cells [177]. Additionally, in the case of hyperglycemia, the protective role of SIRT-1 on the processes of proliferation and migration of endothelial cells of the cerebral microcirculation has been demonstrated in rats [178].

The action of BBR is based mainly on the activation of SIRT1, the synthesis of which is conditioned by the availability of NAD+. BBR is an AMP-activated kinase activator and affects NAD synthesis as it can increase the expression of nicotinamide phosphoribosyltransferase [179]. In atherosclerosis, the promotion of autophagy and apoptosis of peritoneal macrophages through the activation of SIRT1 via the NAD+-mediated EB transcription factor promotion pathway was documented. The process of the regulation of autophagy and apoptosis is crucial in the pathogenesis of atherosclerosis, as it affects the formation and stability of atherosclerotic plaques [180].

BBR has the ability to activate PPARγ through enhancement in the AMPK/SIRT1 pathway. In this mechanism, BBR affects adipose tissue remodeling and thermogenesis [181]. Other mechanisms of action of BBR include modulation in the SIRT1/LKB1/AMPK, SIRT1/PGC-1α, SIRT1/NLRP3, and SIRT3/FoxO pathways. In this mechanism, reduction in doxorubicin cardiotoxicity was found [168]. Based on the available studies, it can be concluded that BBR has a pleiotropic and multidirectional effect, with a significant impact on the activation of signaling pathways involving SIRT1. A summary of the studies describing berberine’s impact on sirtuins is shown in Table 6.

## 15. Kaempferol

Kaempferol (KMP) is an organic chemical compound, a flavonol derivative, widely distributed in the plant world in the form of a glycoside aglycone. It is found in the leaves, seeds, flowers, and fruits of many popular plants, such as cabbage, beans, broccoli, cauliflower, and garlic [182]. KMP has anti-cancer [183,184], cardio- and neuroprotective effects [185], as well as a hepatoprotective effect [186,187]. In the study by Sharma et al., evaluating the role of KMP in triple-negative breast cancer cells (TNBCs), it was shown that this compound inhibits SIRT3 and SIRT6 proteins, which may be a modulator of sirtuin overexpression in TNBCc. This overexpression is significant because it is associated with the biological activity of cancer stem cells, their potential for metastasis, and resistance to chemotherapy [188]. It has been shown that KMP limits liver damage after exposure to acetaminophen. Previous studies evaluating the toxic effects of acetaminophen showed that KMP has a protective effect by suppressing CYP2E1 [187], but in a more recent study, it was shown that the mechanism of action is also based on the activation of SIRT1, resulting in deacetylation of p53, NF-κB and FOXO-1 [186].

In one of the studies on rats, it was shown that KMP reduces the effects of lung ischemia-reperfusion injury (LIRI) by reducing oxidative stress and inhibiting apoptosis by increasing the activity of the SIRT1. In this case, the effects of KMP were associated with the activation of the mitochondrial SIRT1/PGC-1α pathway [189]. In another study evaluating the consequences of LIRI, it was shown that the anti-inflammatory and antioxidant effects of KMP are associated with the activation of the SIRT1/HMGB1/NF-κB pathway [190]. Another rat study evaluating ischemia/reperfusion injury (I/RI) showed a protective effect of KMP by reducing oxidative stress by activating SIRT3 [191], but at the same time, a similar study in rats evaluating cellular effects of I/RI showed a protective effect of KMP on the myocardium by activating SIRT1 [192]. Based on the available literature, it can be concluded that the effect on KMP at the cellular level is based on an anti-inflammatory and antioxidant effect, and the mechanism of action is based mainly on the activation of SIRT1; however, it has been shown that the biological activity of KMP is also associated with the modulation of SIRT3 and SIRT6 activity. A summary of the studies describing kaempferol’s impact on sirtuins is shown in Table 7.

## 16. Conclusions

In this review, SIRT’s involvement in many crucial cellular pathways, especially antioxidant, apoptotic, and anti-inflammatory processes, was presented. It may be assumed that the molecules that modulate their activity may prevent some chronic diseases and are worth further research. Thus, phytochemicals, as a potential group of SIRT’s activators, were summarized. Among them, substances that are commonly found in a variety of plant species were described in this review: resveratrol, curcumin, quercetin, fisetin, berberine, and kaempferol. According to the in vitro and in vivo studies, the abovementioned molecules mainly regulate SIRT1, which influences antioxidant, anti-inflammatory, and antiapoptotic responses. Furthermore, there is evidence that they may regulate fat and carbohydrate metabolism. However, the phytochemical mechanisms of action are multifactorial, and regulation via SIRTs is not the only one. However, additional studies are necessary to verify the efficacy of the mentioned phytochemicals under the conditions specified in the review, particularly in humans.

## Figures and Tables

**Table 1:** Sirtuin activators and their sources [13,28,29,30,58,59,60].

Bioactive Molecules Modulating Sirtuins	Sources
Resveratrol (stilben)	Grapes, red wine, blueberries, cranberries, raisins, peanuts, *Polygonum cuspidatum*
Fisetin (flavonoid)	Apples, grapes, persimmons, strawberries, cucumbers, onion
Curcumin	herbal medicine, dietary spice (from root of the *Curcuma longa*)
Quercetin (flavonoid)	Onion, Shallots, broccoli, peppers, capers, apples, blueberries, grapes, herbs, tea, wine
Berberine	*Coptidis rhizoma*
Honokiol	*Magnolia grandiflora*
Dihydromyricetin (Ampelopsin, flavonool)	Ingredient of the Chinese medicinal herb *Ampelopsis grossedentata*
Trans ε-viniferin (polyphenol stiblenoid)	Vine stalks and most woody parts of the vine
Trilobatin	*Lithocarpus polystahyus Rehd*
Salidroside	Extracted from *Rhodiola rosea*
Silybin	*Silybum marianum (L.) seeds*
Polydatin	*Polygonum cuspidatum*
Kaempferol (flavonoid)	Spinach, kale, herbs, dills, chives, tarragon, wild leeks, ramps
Luteolin (flavonoid)	Carrots, peppers, celery, olive oil, peppermint, thyme, rosemary, lettuce, pomegranate, turnip, capers, cucumber, lemon, beets, brussels sprouts, cabbage, cauliflower, chives, fennel, harwort, horseradish, kohlrabi, parsley, spinach, and green tea
Cyanidin (anthocyanidin)	Berries, black currant, grapes
Delphinidin (anthocyanidin)	Fruits, vegetables, grains
Icariin (prenylated flavonoid glycoside)	*Herba epimedii*
Fucoidan (polysaccharide)	Seaweeds, brown algae
Oleic acid (fatty acid)	Olive oil, nuts vegetable
Linoleic acid (fatty acid)	Plant-based oil, nut, meat, animal products

**Table 2:** Resveratrol: effects and proposed mechanisms of its activity.

Author	Type of Sirtuin	Potential Mechanism of Action
Reinisalo, M. et al., 2015 [63]	SIRT1	deacetylation and activation of liver kinase B1 (LKB1) and ↑AMPK → ↑cellular NAD+ and ↑ catabolism inhibition of the NF-κB -light chain (enhancer of activated B cells) → anti-inflammatory and anti-cancer effects
Vingtdeux, V. et al., 2008 [72]	SIRT1	the anti-amyloidogenic effect of PPARγ activation
Kelly, G.S et al., 2010 [73]	SIRT1	modulation of PPAR γ co-activator 1 α pathway, enhancing mitochondrial function and proteostasis
Thomas, J. et al., 2014 [75]	SIRT1	reduction of pro-inflammatory signaling through Jak-Stat pathway (IL-15, IL-22, Socs2, and Socs5)
Le, K. et al., 2019 [80]	SIRT1	inhibition of HMGB1/TLR4/MyD88/NF-kB signaling and subsequent neuroinflammatory responses, providing neuroprotective effects in neonatal hypoxic-ischemic brain injury
Shen, J. et al., 2018 [81]	SIRT1	activation of SIRT1/miR-134 pathway↑ expression of CREB and BDNF in the hippocampus → prevent impairment of the cognition induced by stress
Cai, M. et al., 2023 [89]	SIRT1	Activation of SIRT1/Nrf2 pathway → ↓ oxidative stress and apoptosis

**Table 3:** Curcumin’s effects and the proposed mechanisms of its activity.

Author	Type of Sirtuin	Potential Mechanism of Action
Feng, K., et al., 2019 [110]	SIRT1	Inhibition of endoplasmic reticulum stress → ↓ osteoarthritis development
Zhang, L., et al., 2019 [111]	SIRT1	protective effects by stimulating mTOR phosphorylation and SIRT1 expression and ↓ the expression of autophagy-related 12, Beclin-1 and microtubule-associated protein light chain 3 II → reduced body weight loss and disease severity
Yin, Y., et al., 2020 [112]	SIRT1	Activation of the SIRT1/Nrf2 pathway and reduced TLR4 expression
Ugur, S., et al., 2015 [113] and Ortega-Domínguez, B. et al., 2017 [114]	SIRT1, SIRT3, SIRT4	cisplatin induced renal impairment → ↓ oxidative stress and ↑ protection of the kidneys from pathological changes
Li, L., et al., 2021 [116]	SIRT1	sepsis induced acute kidney injury increased survival → improved kidney function, reduced inflammatory response and oxidative stress, and prevention of cell apoptosis
He, L., et al., 2015 [117]	SIRT1	protective effects in gentamicin-induced acute kidney injury by ↓ tubular cell apoptosis, oxidative stress and ↑ SIRT1 and Nrf2/HO-1 expression
Jiménez-Flores, L.M., et al., 2014 [119]	SIRT1	Modulation of SIRT1 through AMPK → ↑ glucose absorption and metabolism
Zendedel, E., et al., 2018 [120]	SIRT1	Activation of AMPK, ↑ ATP and superoxide synthesis → ↓ oxidative stress-induced damage to mitochondria and ↓ infarction size
Fusi, J., et al., 2018 [121]	SIRT1	↓ apoptosis and oxidative stress
Li, K., et al., 2019 [122]	SIRT1	streptozotocin-induced diabetes, ↑ cardiac function, ↓ myocardial fibrosis and cardiac hypertrophy, ↓ ROS generation
Takano, K., et al., 2018 [127]	SIRT1	Long-term administration in atherosclerosis induced by a high-fat diet protected against ↓ SIRT1 expression, senescent cell accumulation, and vascular inflammation
Sun, Q., et al., 2014 [132]	SIRT1	↓ the expression of Bax a protein involved in apoptosis, in the presence of Aβ25-35
Jia, N., et al., 2016 [133]	SIRT1	↓ glutamate excitotoxicity in cultured neurons and ↓ the level of acetylated PGC1α through deacetylation
Zhang, M. et al., 2017 [107]	SIRT3	↑ expression of SIRT3 in COPD → ↓ oxidative stress and inflammation markers
Liu, M., et al., 2021 [131]	SIRT3	↑ SIRT3 expression → ↓ superoxide dismutase activity, ↓ oxidative stress, and ↓ iron loading-induced autophagy in cell models of iron overload
Tan, C., et al., 2021 [125] and Lin, X., et al., 2015 [126]	SIRT6	↓ foam cell formation and intracellular lipid accumulation, ↑ cholesterol efflux in macrophages and activation of AMPK-SIRT1-LXRα signaling pathway → anti-atherosclerotic effects

**Table 4:** Quercetin’s effects and the proposed mechanisms of its activity.

Author	Type of Sirtuin	Potential Mechanism of Action
Cui Z et al., 2022 [149]	SIRT1	regulation of pathways: SIRT1/AMPK/NFκB, SIRT1/Keap1/Nrf2/HO-1 and SIRT1/PI3K/Akt → ↑ the activity of antioxidant enzymes and anti-inflammatory cytokines
Guo H. et al., 2021 [153]	SIRT1	inhibition of oxLDL-induced mitochondrial dysfunction and ROS production
Sang A. et al., 2022 [154]	SIRT1	↓ expression mRNA of CHOP, GRP78, activation of transcription factor 6 (ATF6), ↑ activation of AMPK/SIRT1 pathway → prevention of mitochondrial dysfunction and suppression of oxidative stress
Feng K. et al., 2019 [161]	SIRT1	↓ chondrocytes apoptosis by attenuation of ER stress and downregulation of the factors: CHOP, GRP78 and caspase 3
Tang J. et al., 2019 [158]	SIRT1	SIRT1/PGC-1α signaling pathway → up-regulation Bcl-2 and down-regulation Bax expression → anti-apoptotic impact in ischemia-reperfusion injury
Yang R. et al., 2022 [160]	SIRT1	NRF2/HO-1 pathway → ↓ ROS production in the mitochondria in ischemia-reperfusion injured brain cells

**Table 5:** Fisetin’s effects and the proposed mechanisms of its activity.

Author	Type of Sirtuin	Potential Mechanism of Action
Kim S.C. et al., 2015 [162]	SIRT1	↓ the binding of PPARγ to the PPARγ promotor and simultaneously ↑ the binding of SIRT1 to the promoter → possibly changes deacetylation and activity of PPARγ → ↓ adipogenesis and accumulation of lipids control of adipogenic transcription factors such as CCAAT enhancer binding protein (C/EBP) family, peroxisome proliferator-activated receptor gamma (PPARg), Krüppel-like factors (KLFs) and sterol regulatory element-binding protein 1c (SREBP1c) → induction of adipocyte differentiation
Singh S., et al., 2020 [164] and Singh S., et al., 2018 [165]	SIRT1	enhanced NF-kB deacetylation and suppression of pro-inflammatory genes expression in brain cells → prevention of neuro-inflammation and natural aging
Zheng W. et al., 2017 [166]	SIRT1	inhibition of IL-1β-induced inflammation and degradation of Sox-9, aggrecan and collagen-II → ↓ articular cartilage damage, subchondral bone sclerosis and synovitis
Rizk F.H., et al., 2022 [167]	SIRT1	degradation of the transcription factor FOXO3a → ↓ apoptosis

**Table 6:** Berberine’s effects and the proposed mechanisms of its activity.

Author	Type of Sirtuin	Potential Mechanism of Action
Yu L. et al., 2016 [174]	SIRT1	↑ expression of the antiapoptotic factor Bcl-2 and ↓ expression in the proapoptotic factors Bax and caspase-
Xue Y. et al., 2020 [175]	SIRT1	downregulation of gp91^phox^ → antioxidant and anti-inflammatory effect ↓activity of IL-1β, IL-6, and TNFα → antioxidant and anti-inflammatory effect
El-Zeftawy, M. et al., 2019 [177]	SIRT1	PI3K/Akt-p/SIRT-1/PTEN pathway → ↓ insulin resistance
Xu, Y. et al., 2021 [181]	SIRT1	AMPK/SIRT1 pathway → activation of PPARγ → tissue remodeling and thermogenesis
Tabrizi, F.B. et al., 2022 [168]	SIRT1	SIRT1/LKB1/AMPK, SIRT1/PGC-1α, SIRT1/NLRP3 and SIRT3/FoxO pathways → ↓ doxorubicin cardiotoxicity

**Table 7:** Kaempferol and the effects and proposed mechanisms of its activity.

Author	Type of Sirtuin	Potential Mechanism of Action
BinMowyna, M.N. et al., 2021 [186]	SIRT1	exposure to acetaminophen → deacetylation of p53, NF-κB and FOXO-1 → hepatoprotective effect
Yang, C., et al., 2021 [189]	SIRT1	activation of the mitochondrial SIRT1/PGC-1α pathway → ↓ oxidative stress and apoptosis
Yang, C., et al., 2019 [190]	SIRT1	activation of the SIRT1/HMGB1/NF-κB pathway → anti-inflammatory and antioxidant effects
Guo, Z., et al., 2015 [192]	SIRT1	↓ LDH release in cardiomyoytes, ROS, caspase-3 and apoptosis and ↑ Bcl2
Sun, C., et al., 2022 [191]	SIRT3	↓ ROS, NADPH oxidase activity, Bax and ↑ glutathione, Bcl2 → reduced oxidative stress

## Data Availability

Not applicable.
